# Crystal structure of bis­[μ-1,4-bis­(di­phenyl­phos­phan­yl)butane-κ^2^ *P*:*P*′]bis­[(3,4,7,8-tetra­methyl-1,10-phenanthroline-κ^2^ *N*,*N*′)copper(I)] bis­(hexa­fluorido­phosphate) di­chloro­methane disolvate

**DOI:** 10.1107/S2056989016015553

**Published:** 2016-10-11

**Authors:** Michihiro Nishikawa, Kotaro Mutsuura, Taro Tsubomura

**Affiliations:** a Department of Materials and Life Science, Seikei University, 3-3-1 Kichijoji-kitamachi, Musashino-shi, Tokyo, Japan

**Keywords:** crystal structure, copper(I) complexes, diphosphines, di­imine, π–π inter­actions

## Abstract

The dication of the title compound, [Cu_2_(C_28_H_28_P_2_)_2_(C_16_H_16_N_2_)_2_](PF_6_)_2_·2CH_2_Cl_2_, has crystallographically imposed inversion symmetry. The copper(I) cation is coordinated in a distorted tetra­hedral geometry by two N atoms of a chelating 3,4,7,8-tetra­methyl-1,10-phenanthroline ligand and two P atoms of two bridging 1,4-bis­(di­phenyl­phosphan­yl)butane ligands, forming a 14-membered ring. An intra­molecular π–π inter­action stabilizes the conformation of the dication. In the crystal, dications are linked by π–π inter­actions involving adjacent phenanthroline rings, forming chains running parallel to [111]. Weak C—H⋯F hydrogen inter­actions are also observed.

## Abstract 2

The crystal structure of a dinuclear copper(I) complex bearing bridging 1,4-bis­(di­phenyl­phosphan­yl)butane and 3,4,7,8-tetra­methyl-1,10-phenanthroline ligands is described.

## Chemical context   

Copper(I) complexes bearing di­imine ligands are important candidates for photofunctional materials due to the possible generation of long-lived charge-transfer excited states (Barbieri *et al.*, 2008 ▸; Nishikawa *et al.*, 2015 ▸). We have previously reported the crystal structures as well as the long-lived emission properties of the dicopper(I) complexes [Cu_2_(dmp)_2_(dppb)_2_](PF_6_)_2_ (dppb = 1,4-bis­(di­phenyl­phos­phan­yl)butane, dmp = 2,9-dimethyl-1,10-phenanthroline) (Saito *et al.*, 2006 ▸) and [Cu_2_(dmpp)_2_(dppb)_2_](PF_6_)_2_ (dmpp = 4,7-diphenyl-1,10-phenanthroline) (Tsubomura *et al.*, 2015 ▸). In addition, the synthesis and NMR studies of dicopper(I) complexes bearing 1,1-bis­(di­phenyl­phosphan­yl)methane and 3,4,7,8-tetra­methyl-1,10-phenanthroline (tmp) ligands (Kitagawa *et al.*, 1991 ▸), and the crystal structures of bis­(di­imine)­copper(I) complexes, [Cu(tmp)_2_]BPh_4_ and [Cu(phen)_2_]BPh_4_ (Cunningham *et al.*, 2000 ▸), have been reported. It is known that methyl substitution on the phenanthroline ligand often gives the essential effect on the photophysical properties of the copper complexes. Herein we describe the synthesis and crystal structure of a novel dinuclear copper(I) complex bearing tmp and dppb ligands. The title complex, [Cu_2_(tmp)_2_(dppb)_2_](PF_6_)_2_·2CH_2_Cl_2_, was newly synthesized by the reaction of tmp, dppb, and tetra­kis­(aceto­nitrile)­copper(I) hexa­fluorido­phosphate in di­chloro­methane at room temperature.

## Structural commentary   

**Figure 1:**
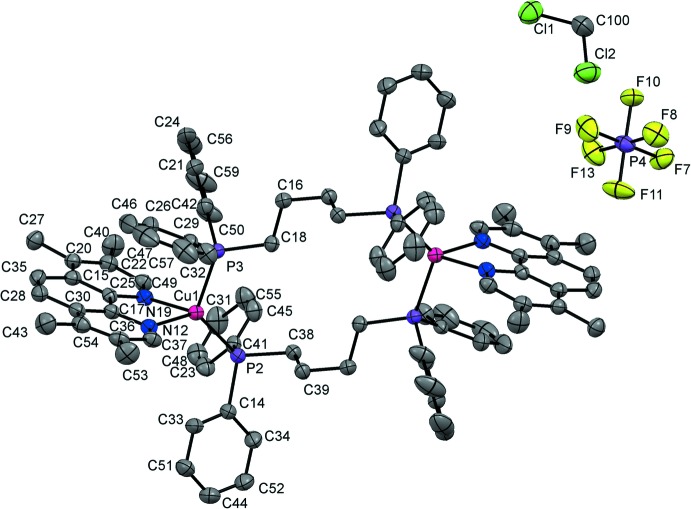
The mol­ecular structure of the title compound, with displacement ellipsoids drawn at the 50% probability level. Unlabelled atoms are related to the labelled atoms by (−*x*, −*y*, −*z*). H atoms have been omitted for clarity.

The asymmetric unit of the title compound consists of half of the dicopper(I) complex cation, one hexa­fluorido­phosphate counter-anion, and one di­chloro­methane mol­ecule. The complex has crystallographically imposed inversion symmetry. Each copper(I) atom is coordinated in a distorted tetra­hedral geometry by two nitro­gen atoms of a chelating tmp mol­ecule and two phospho­rus atoms of two centrosymmetric bridging dppb ligands, forming a 14-membered ring (Fig. 1 ▸).
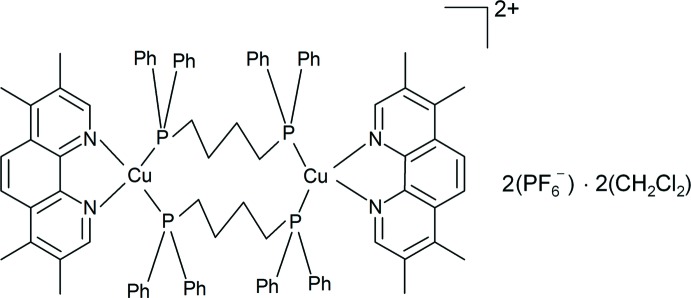


The distorted tetra­hedral geometry around the copper(I) cation is characteristic of copper(I) complexes bearing di­imine and diphosphine ligands. The Cu—N bond lengths [2.063 (4) and 2.091 (4) Å] are shorter than those observed in the related complexes [Cu_2_(dmpp)_2_(dppb)_2_](PF_6_)_2_ [2.080 (4) and 2.130 (4) Å] and [Cu_2_(dmp)_2_(dppb)_2_](PF_6_)_2_ [2.105 (4) and 2.117 (4) Å]. The Cu—P bonds [2.212 (2) and 2.276 (2) Å] are also shorter than those of [Cu_2_(dmpp)_2_(dppb)_2_](PF_6_)_2_ [2.2669 (15) and 2.2915 (16) Å] and [Cu_2_(dmp)_2_(dppb)_2_] [2.256 (1) and 2.3002 (14) Å]. The N—Cu—N bond angle of 80.10 (13)° is not significantly different from those of [Cu_2_(dmpp)_2_(dppb)_2_](PF_6_)_2_ [80.03 (14)°] and [Cu_2_(dmp)_2_(dppb)_2_](PF_6_)_2_ [80.1 (2)°], whereas the P—Cu—P bond angle [122.83 (8)°] falls in the range observed for [Cu_2_(dmpp)_2_(dppb)_2_](PF_6_)_2_ [119.57 (5)°] and [Cu_2_(dmp)_2_(dppb)_2_](PF_6_)_2_ [126.38 (5)°]. The conformation of the dinuclear complex is stabilized by the presence of two relatively short intramolecular π–π inter­actions involving the N12/C17/C30/C54/C36/C37 pyridine ring and the C29/C26/C46/C47/C57/C32 phenyl ring of the dppb ligand [centroid-to-centroid distance = 3.577 (5) Å].

## Supra­molecular features   

**Figure 2:**
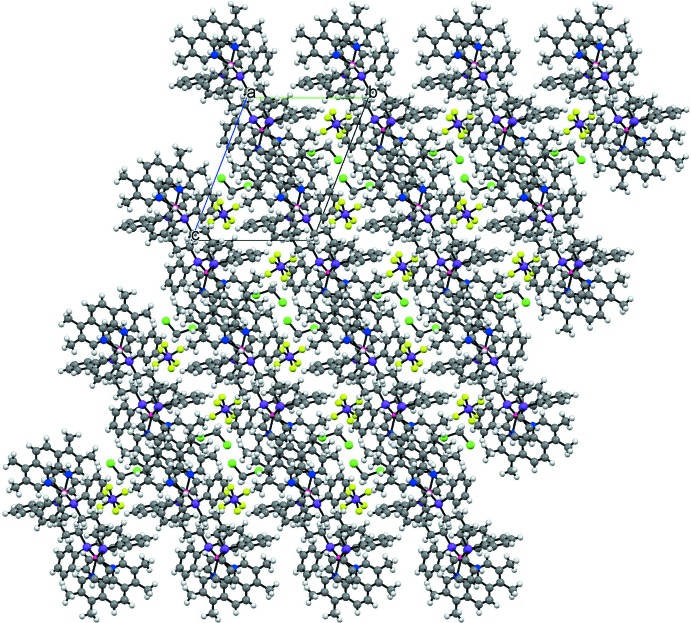
The packing of the title compound, viewed along the *a* axis.

In the crystal, π–π inter­actions between the phenanthroline rings of adjacent complex dications are observed [centroid-to-centroid distance = 3.644 (4) Å], forming chains running parallel to [111]. As shown in Fig. 2 ▸, the di­chloro­methane solvent mol­ecules and counter-ions are sandwiched by the chains of the complex cations. There are weak inter­molecular C—H⋯F hydrogen-bonding inter­actions between the fluorine atoms of the counter-ion and the methyl­ene group of the di­chloro­methane mol­ecule. An inter­molecular C—H⋯F hydrogen bond involving an aromatic C—H group of a phenyl ring is also observed (Table 1 ▸). Inter­molecular π–π inter­actions between phenanthroline rings are not observed in the crystal structure of [Cu_2_(dmp)_2_(dppb)_2_](PF_6_)_2_, where only weak intra­molecular inter­actions are present between the phenanthroline ring and the phenyl rings of the diphosphine moieties.

## Synthesis and crystallization   

Under an argon atmosphere, [Cu(MeCN)_4_]PF_6_ (75 mg, 0.20 mmol) was added to a CH_2_Cl_2_ solution of dppb (85 mg, 0.20 mmol). Then, tmp (45 mg, 0.20 mmol) was added and the reaction mixture was stirred for 100 min at room temperature. After addition of *n-*hexane to the solution, the formed solid was filtered, washed with diethyl ether, and dried *in vacuo* (yield; 139 mg, 80%). Single crystals of the title compound suitable for X-ray analysis were obtained by slow diffusion of diethyl ether into the di­chloro­methane solution.

## Refinement   

Data collection details and refinement results are summarized in Table 2 ▸. All H atoms were positioned geometrically and refined using a riding model with C—H = 0.99 Å and *U*_iso_(H) = 1.2*U*_eq_(C) for methyl­ene groups, C—H = 0.98 Å and *U*_iso_(H) = 1.2*U*_eq_(C) for the methyl groups and C—H = 0.95 Å and *U*_iso_(H) = 1.2*U*_eq_(C) for the aromatic groups. A rotation model was used for the methyl groups.

## Supplementary Material

Crystal structure: contains datablock(s) global, I. DOI: 10.1107/S2056989016015553/rz5194sup1.cif

Structure factors: contains datablock(s) I. DOI: 10.1107/S2056989016015553/rz5194Isup2.hkl

CCDC reference: 1507981

Additional supporting information: crystallographic information; 3D view; checkCIF report

## Figures and Tables

**Table 1:** Hydrogen-bond geometry (Å, °)

*D*—H⋯*A*	*D*—H	H⋯*A*	*D*⋯*A*	*D*—H⋯*A*
C32—H32⋯F10^i^	0.95	2.51	3.382 (6)	152
C100—H10*A*⋯F11^ii^	0.99	2.39	3.360 (8)	165
C100—H10*A*⋯F13^ii^	0.99	2.55	3.373 (9)	141

Symmetry codes: (i) 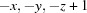; (ii) 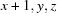.

**Table 2:** Experimental details

Crystal data	
Chemical formula	[Cu_2_(C_28_H_28_P_2_)_2_(C_16_H_16_N_2_)_2_](PF_6_)_2_·2CH_2_Cl_2_
*M*_r_	1912.38
Crystal system, space group	Triclinic, *P* 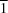
Temperature (K)	123
*a*, *b*, *c* (Å)	11.723 (15), 12.967 (16), 16.06 (2)
α, β, γ (°)	108.302 (13), 98.665 (12), 103.284 (13)
*V* (Å^3^)	2190 (5)
*Z*	1
Radiation type	Mo *K*α
μ (mm^−1^)	0.79
Crystal size (mm)	0.5 × 0.5 × 0.2

Data collection	
Diffractometer	Rigaku Saturn70 CCD
Absorption correction	Multi-scan (*REQAB*; Rigaku, 1998 ▸)
*T*_min_, *T*_max_	0.892, 1
No. of measured, independent and observed [*I* > 2σ(*I*)] reflections	20388, 9329, 6951
*R*_int_	0.044
(sin θ/λ)_max_ (Å^−1^)	0.649

Refinement	
*R*[*F* ^2^ > 2σ(*F* ^2^)], *wR*(*F* ^2^), *S*	0.072, 0.169, 1.09
No. of reflections	9329
No. of parameters	536
H-atom treatment	H-atom parameters constrained
Δρ_max_, Δρ_min_ (e Å^−3^)	0.62, −0.52

Computer programs: *CrystalClear* (Rigaku, 2000 ▸), *SIR92* (Altomare *et al.*, 1994 ▸), *SHELXL97* (Sheldrick, 2008 ▸), *Mercury* (Macrae *et al.*, 2008 ▸) and *publCIF* (Westrip, 2010 ▸).

## supplementary crystallographic information

### Crystal data

**Table:**

[Cu_2_(C_28_H_28_P_2_)_2_(C_16_H_16_N_2_)_2_](PF_6_)_2_·2CH_2_Cl_2_	*Z* = 1
*M**_r_* = 1912.38	*F*(000) = 984
Triclinic, *P*1	*D*_x_ = 1.45 Mg m^−^^3^
*a* = 11.723 (15) Å	Mo *K*α radiation, λ = 0.71073 Å
*b* = 12.967 (16) Å	Cell parameters from 5584 reflections
*c* = 16.06 (2) Å	θ = 3.2–27.5°
α = 108.302 (13)°	µ = 0.79 mm^−^^1^
β = 98.665 (12)°	*T* = 123 K
γ = 103.284 (13)°	Block, yellow
*V* = 2190 (5) Å^3^	0.5 × 0.5 × 0.2 mm

### Data collection

**Table:**

Rigaku Saturn70 CCD diffractometer	6951 reflections with *I* > 2σ(*I*)
dtprofit.ref scans	*R*_int_ = 0.044
Absorption correction: multi-scan (*REQAB*; Rigaku, 1998)	θ_max_ = 27.5°, θ_min_ = 3.2°
*T*_min_ = 0.892, *T*_max_ = 1	*h* = −13→15
20388 measured reflections	*k* = −16→16
9329 independent reflections	*l* = −20→20

### Refinement

**Table:**

Refinement on *F*^2^	0 restraints
Least-squares matrix: full	H-atom parameters constrained
*R*[*F*^2^ > 2σ(*F*^2^)] = 0.072	*w* = 1/[σ^2^(*F*_o_^2^) + (0.0723*P*)^2^ + 2.0278*P*] where *P* = (*F*_o_^2^ + 2*F*_c_^2^)/3
*wR*(*F*^2^) = 0.169	(Δ/σ)_max_ = 0.013
*S* = 1.09	Δρ_max_ = 0.62 e Å^−^^3^
9329 reflections	Δρ_min_ = −0.52 e Å^−^^3^
536 parameters	

### Special details

**Table:**

Geometry. All esds (except the esd in the dihedral angle between two l.s. planes) are estimated using the full covariance matrix. The cell esds are taken into account individually in the estimation of esds in distances, angles and torsion angles; correlations between esds in cell parameters are only used when they are defined by crystal symmetry. An approximate (isotropic) treatment of cell esds is used for estimating esds involving l.s. planes.

### Fractional atomic coordinates and isotropic or equivalent isotropic displacement parameters (Å^2^)

**Table:**

	*x*	*y*	*z*	*U*_iso_*/*U*_eq_	
Cu1	0.26867 (4)	0.23663 (4)	0.22973 (3)	0.02662 (15)	
P2	0.36165 (9)	0.12357 (9)	0.15197 (7)	0.0275 (2)	
P3	0.09667 (9)	0.26348 (8)	0.16520 (7)	0.0265 (2)	
P4	0.12149 (11)	0.18077 (11)	0.81650 (8)	0.0395 (3)	
F7	0.0743 (3)	0.2452 (3)	0.8990 (2)	0.0573 (8)	
F8	0.2540 (3)	0.2682 (3)	0.8601 (2)	0.0655 (9)	
F9	0.1681 (3)	0.1150 (3)	0.7334 (2)	0.0746 (10)	
F10	0.1578 (3)	0.1026 (2)	0.8698 (2)	0.0572 (8)	
F11	0.0859 (3)	0.2600 (3)	0.7650 (2)	0.0701 (10)	
N12	0.2443 (3)	0.2589 (3)	0.3581 (2)	0.0292 (7)	
F13	−0.0099 (3)	0.0909 (3)	0.7726 (2)	0.0702 (9)	
C14	0.4484 (4)	0.0520 (3)	0.2078 (3)	0.0310 (9)	
C15	0.4132 (3)	0.5631 (3)	0.4366 (3)	0.0296 (9)	
C16	−0.1100 (4)	0.1833 (3)	0.0189 (3)	0.0312 (9)	
H16A	−0.0815	0.252	0.0042	0.037*	
H16B	−0.1577	0.2015	0.0646	0.037*	
C17	0.2907 (3)	0.3699 (3)	0.4152 (3)	0.0281 (8)	
C18	−0.0006 (3)	0.1546 (3)	0.0598 (3)	0.0284 (8)	
H18A	0.0486	0.1401	0.0148	0.034*	
H18B	−0.0297	0.0833	0.0708	0.034*	
N19	0.3846 (3)	0.4014 (3)	0.2999 (2)	0.0293 (7)	
C20	0.4835 (4)	0.6354 (3)	0.4000 (3)	0.0314 (9)	
C21	0.0450 (4)	0.4616 (4)	0.1457 (3)	0.0414 (11)	
H21	−0.0273	0.4413	0.1645	0.05*	
C22	0.5032 (4)	0.5885 (4)	0.3153 (3)	0.0335 (9)	
C23	0.5963 (4)	0.2594 (4)	0.1741 (3)	0.0387 (10)	
H23	0.6189	0.236	0.223	0.046*	
C24	0.0731 (6)	0.5596 (4)	0.1243 (3)	0.0556 (15)	
H24	0.0205	0.6059	0.1287	0.067*	
C25	0.3660 (3)	0.4468 (3)	0.3833 (3)	0.0254 (8)	
C26	0.0186 (5)	0.3783 (4)	0.3143 (3)	0.0446 (12)	
H26	0.0771	0.445	0.3182	0.053*	
C27	0.5335 (4)	0.7615 (3)	0.4518 (3)	0.0395 (10)	
H27A	0.6038	0.7766	0.5003	0.059*	
H27B	0.4714	0.79	0.4779	0.059*	
H27C	0.558	0.8	0.4109	0.059*	
C28	0.3189 (4)	0.5288 (4)	0.5536 (3)	0.0356 (10)	
H28	0.3056	0.5576	0.6123	0.043*	
C29	−0.0020 (4)	0.2758 (3)	0.2432 (3)	0.0298 (9)	
C30	0.2647 (4)	0.4102 (4)	0.5002 (3)	0.0317 (9)	
C31	0.6490 (5)	0.3739 (5)	0.0858 (4)	0.0545 (14)	
H31	0.7068	0.4299	0.0752	0.065*	
C32	−0.0873 (4)	0.1812 (4)	0.2409 (3)	0.0420 (11)	
H32	−0.1021	0.1107	0.1931	0.05*	
C33	0.4536 (4)	0.0697 (4)	0.2980 (3)	0.0388 (10)	
H33	0.4097	0.1155	0.3294	0.047*	
C34	0.5091 (4)	−0.0187 (4)	0.1620 (3)	0.0409 (11)	
H34	0.5048	−0.0327	0.0998	0.049*	
C35	0.3884 (4)	0.6012 (4)	0.5232 (3)	0.0338 (9)	
H35	0.4213	0.6792	0.5607	0.041*	
C36	0.1367 (4)	0.2211 (4)	0.4677 (3)	0.0357 (10)	
C37	0.1683 (4)	0.1886 (4)	0.3844 (3)	0.0320 (9)	
H37	0.1335	0.1119	0.3443	0.038*	
C38	0.2725 (4)	0.0082 (3)	0.0463 (3)	0.0290 (9)	
H38A	0.2218	0.0378	0.0101	0.035*	
H38B	0.3273	−0.022	0.0107	0.035*	
C39	0.1922 (4)	−0.0868 (3)	0.0665 (3)	0.0312 (9)	
H39A	0.1415	−0.0548	0.1056	0.037*	
H39B	0.2438	−0.1182	0.1004	0.037*	
C40	0.5725 (5)	0.6565 (4)	0.2687 (4)	0.0494 (12)	
H40A	0.5233	0.699	0.2472	0.074*	
H40B	0.5919	0.605	0.2172	0.074*	
H40C	0.6474	0.7098	0.3113	0.074*	
C41	0.4780 (4)	0.2122 (3)	0.1195 (3)	0.0313 (9)	
C42	0.1216 (4)	0.3938 (3)	0.1397 (3)	0.0345 (10)	
C43	0.1520 (5)	0.3689 (4)	0.6160 (3)	0.0496 (12)	
H43A	0.0786	0.3925	0.6088	0.074*	
H43B	0.2182	0.4327	0.6601	0.074*	
H43C	0.1376	0.3051	0.6372	0.074*	
C44	0.5851 (4)	−0.0473 (4)	0.2978 (3)	0.0454 (12)	
H44	0.6341	−0.0789	0.3292	0.054*	
C45	0.4481 (4)	0.2465 (4)	0.0471 (3)	0.0404 (10)	
H45	0.3687	0.2151	0.0091	0.048*	
C46	−0.0477 (6)	0.3818 (6)	0.3797 (3)	0.0634 (17)	
H46	−0.0342	0.4516	0.4279	0.076*	
C47	−0.1324 (5)	0.2861 (7)	0.3756 (4)	0.0668 (18)	
H47	−0.1767	0.2899	0.4208	0.08*	
C48	0.6798 (4)	0.3394 (4)	0.1573 (3)	0.0478 (12)	
H48	0.7593	0.3712	0.1951	0.057*	
C49	0.4531 (4)	0.4712 (4)	0.2693 (3)	0.0334 (9)	
H49	0.4692	0.439	0.2121	0.04*	
C50	0.2252 (4)	0.4237 (5)	0.1098 (4)	0.0496 (13)	
H50	0.2777	0.3773	0.1038	0.06*	
C51	0.5230 (5)	0.0205 (4)	0.3430 (3)	0.0460 (12)	
H51	0.5273	0.034	0.4052	0.055*	
C52	0.5765 (4)	−0.0695 (4)	0.2073 (3)	0.0445 (11)	
H52	0.6164	−0.1194	0.1754	0.053*	
C53	0.0479 (5)	0.1334 (4)	0.4881 (4)	0.0500 (13)	
H53A	0.0865	0.1228	0.5421	0.075*	
H53B	0.0222	0.0611	0.4367	0.075*	
H53C	−0.0226	0.1593	0.4989	0.075*	
C54	0.1851 (4)	0.3328 (4)	0.5270 (3)	0.0354 (10)	
C55	0.5341 (5)	0.3265 (4)	0.0301 (3)	0.0516 (13)	
H55	0.5135	0.3485	−0.0199	0.062*	
C56	0.1779 (6)	0.5883 (5)	0.0967 (4)	0.0689 (18)	
H56	0.1981	0.6555	0.0831	0.083*	
C57	−0.1519 (5)	0.1869 (6)	0.3069 (4)	0.0593 (15)	
H57	−0.2103	0.1206	0.3038	0.071*	
C59	0.2525 (5)	0.5213 (6)	0.0887 (4)	0.0709 (19)	
H59	0.3237	0.5413	0.0686	0.085*	
Cl1	0.74499 (14)	0.04960 (12)	0.56451 (11)	0.0641 (4)	
Cl2	0.79599 (14)	0.29365 (12)	0.61857 (12)	0.0734 (5)	
C100	0.7937 (6)	0.1792 (5)	0.6560 (4)	0.0699 (17)	
H10A	0.8757	0.1894	0.6901	0.084*	
H10B	0.7388	0.1784	0.6971	0.084*	

### Atomic displacement parameters (Å^2^)

**Table:**

	*U*^11^	*U*^22^	*U*^33^	*U*^12^	*U*^13^	*U*^23^
Cu1	0.0277 (3)	0.0262 (3)	0.0270 (3)	0.0094 (2)	0.0062 (2)	0.0102 (2)
P2	0.0279 (5)	0.0278 (5)	0.0257 (6)	0.0101 (4)	0.0039 (4)	0.0081 (4)
P3	0.0272 (5)	0.0249 (5)	0.0277 (6)	0.0090 (4)	0.0051 (4)	0.0097 (4)
P4	0.0404 (7)	0.0554 (7)	0.0350 (7)	0.0234 (6)	0.0142 (5)	0.0237 (6)
F7	0.082 (2)	0.0625 (18)	0.0543 (18)	0.0418 (17)	0.0413 (16)	0.0306 (15)
F8	0.0492 (18)	0.073 (2)	0.075 (2)	0.0079 (16)	0.0152 (16)	0.0347 (18)
F9	0.072 (2)	0.115 (3)	0.0448 (19)	0.049 (2)	0.0251 (16)	0.0196 (19)
F10	0.074 (2)	0.0507 (17)	0.0560 (18)	0.0306 (15)	0.0092 (15)	0.0257 (14)
F11	0.071 (2)	0.113 (3)	0.077 (2)	0.053 (2)	0.0374 (18)	0.074 (2)
N12	0.0282 (17)	0.0286 (17)	0.0306 (19)	0.0055 (14)	0.0055 (14)	0.0132 (14)
F13	0.0462 (18)	0.091 (2)	0.062 (2)	0.0077 (17)	0.0022 (15)	0.0260 (18)
C14	0.034 (2)	0.032 (2)	0.028 (2)	0.0137 (18)	0.0034 (17)	0.0101 (17)
C15	0.027 (2)	0.030 (2)	0.033 (2)	0.0097 (17)	0.0049 (17)	0.0134 (18)
C16	0.034 (2)	0.028 (2)	0.031 (2)	0.0119 (17)	0.0046 (18)	0.0101 (17)
C17	0.026 (2)	0.029 (2)	0.030 (2)	0.0073 (16)	0.0031 (16)	0.0137 (17)
C18	0.030 (2)	0.029 (2)	0.026 (2)	0.0096 (17)	0.0081 (17)	0.0089 (16)
N19	0.0292 (18)	0.0302 (17)	0.0294 (19)	0.0062 (14)	0.0080 (14)	0.0133 (15)
C20	0.030 (2)	0.029 (2)	0.034 (2)	0.0090 (17)	0.0036 (18)	0.0123 (18)
C21	0.047 (3)	0.032 (2)	0.040 (3)	0.015 (2)	−0.001 (2)	0.009 (2)
C22	0.029 (2)	0.035 (2)	0.037 (2)	0.0078 (18)	0.0073 (18)	0.0169 (19)
C23	0.026 (2)	0.046 (3)	0.041 (3)	0.0103 (19)	0.0071 (19)	0.014 (2)
C24	0.081 (4)	0.033 (2)	0.045 (3)	0.023 (3)	−0.010 (3)	0.011 (2)
C25	0.0186 (18)	0.030 (2)	0.026 (2)	0.0062 (15)	0.0037 (15)	0.0097 (16)
C26	0.056 (3)	0.046 (3)	0.032 (3)	0.026 (2)	0.006 (2)	0.008 (2)
C27	0.042 (3)	0.030 (2)	0.043 (3)	0.0074 (19)	0.008 (2)	0.0111 (19)
C28	0.038 (2)	0.039 (2)	0.030 (2)	0.017 (2)	0.0061 (19)	0.0095 (19)
C29	0.029 (2)	0.036 (2)	0.023 (2)	0.0149 (18)	0.0021 (16)	0.0078 (17)
C30	0.032 (2)	0.036 (2)	0.030 (2)	0.0130 (18)	0.0037 (17)	0.0146 (18)
C31	0.041 (3)	0.056 (3)	0.055 (3)	−0.008 (2)	0.019 (2)	0.016 (3)
C32	0.036 (2)	0.051 (3)	0.039 (3)	0.007 (2)	0.011 (2)	0.020 (2)
C33	0.049 (3)	0.040 (2)	0.034 (2)	0.025 (2)	0.007 (2)	0.015 (2)
C34	0.041 (3)	0.046 (3)	0.035 (3)	0.023 (2)	0.006 (2)	0.007 (2)
C35	0.034 (2)	0.032 (2)	0.032 (2)	0.0124 (18)	0.0074 (18)	0.0068 (18)
C36	0.034 (2)	0.041 (2)	0.041 (3)	0.0134 (19)	0.0115 (19)	0.025 (2)
C37	0.032 (2)	0.033 (2)	0.036 (2)	0.0077 (18)	0.0079 (18)	0.0204 (19)
C38	0.031 (2)	0.030 (2)	0.025 (2)	0.0089 (17)	0.0034 (16)	0.0099 (16)
C39	0.035 (2)	0.030 (2)	0.027 (2)	0.0078 (18)	0.0039 (17)	0.0099 (17)
C40	0.053 (3)	0.040 (3)	0.055 (3)	0.002 (2)	0.019 (2)	0.023 (2)
C41	0.033 (2)	0.031 (2)	0.028 (2)	0.0098 (18)	0.0094 (17)	0.0070 (17)
C42	0.035 (2)	0.031 (2)	0.032 (2)	0.0051 (18)	0.0007 (18)	0.0095 (18)
C43	0.060 (3)	0.057 (3)	0.042 (3)	0.021 (3)	0.024 (2)	0.024 (2)
C44	0.050 (3)	0.044 (3)	0.047 (3)	0.023 (2)	0.005 (2)	0.020 (2)
C45	0.037 (2)	0.042 (2)	0.035 (3)	0.003 (2)	0.009 (2)	0.010 (2)
C46	0.084 (4)	0.088 (4)	0.030 (3)	0.063 (4)	0.014 (3)	0.009 (3)
C47	0.057 (4)	0.124 (6)	0.050 (4)	0.056 (4)	0.029 (3)	0.044 (4)
C48	0.035 (3)	0.056 (3)	0.047 (3)	0.010 (2)	0.011 (2)	0.013 (2)
C49	0.035 (2)	0.035 (2)	0.033 (2)	0.0093 (18)	0.0092 (18)	0.0173 (19)
C50	0.038 (3)	0.064 (3)	0.061 (3)	0.011 (2)	0.010 (2)	0.045 (3)
C51	0.061 (3)	0.052 (3)	0.034 (3)	0.028 (3)	0.008 (2)	0.020 (2)
C52	0.045 (3)	0.040 (3)	0.048 (3)	0.022 (2)	0.008 (2)	0.010 (2)
C53	0.054 (3)	0.048 (3)	0.058 (3)	0.009 (2)	0.027 (3)	0.031 (3)
C54	0.035 (2)	0.043 (2)	0.034 (2)	0.014 (2)	0.0111 (19)	0.019 (2)
C55	0.056 (3)	0.057 (3)	0.039 (3)	0.003 (3)	0.013 (2)	0.023 (2)
C56	0.085 (5)	0.047 (3)	0.066 (4)	−0.001 (3)	−0.011 (3)	0.036 (3)
C57	0.038 (3)	0.098 (5)	0.053 (3)	0.018 (3)	0.018 (2)	0.041 (3)
C59	0.051 (3)	0.084 (4)	0.090 (5)	0.002 (3)	0.002 (3)	0.068 (4)
Cl1	0.0641 (9)	0.0491 (7)	0.0746 (10)	0.0129 (7)	0.0155 (7)	0.0197 (7)
Cl2	0.0685 (10)	0.0511 (8)	0.0955 (12)	0.0158 (7)	0.0000 (8)	0.0303 (8)
C100	0.087 (5)	0.064 (4)	0.053 (4)	0.028 (3)	−0.006 (3)	0.020 (3)

### Geometric parameters (Å, º)

**Table:**

Cu1—N12	2.063 (4)	C31—C55	1.380 (7)
Cu1—N19	2.091 (4)	C31—C48	1.386 (8)
Cu1—P2	2.212 (2)	C31—H31	0.95
Cu1—P3	2.276 (2)	C32—C57	1.387 (7)
P2—C41	1.823 (4)	C32—H32	0.95
P2—C38	1.834 (4)	C33—C51	1.394 (6)
P2—C14	1.839 (4)	C33—H33	0.95
P3—C29	1.824 (4)	C34—C52	1.397 (6)
P3—C18	1.827 (4)	C34—H34	0.95
P3—C42	1.831 (5)	C35—H35	0.95
P4—F7	1.580 (3)	C36—C54	1.386 (6)
P4—F9	1.589 (3)	C36—C37	1.404 (6)
P4—F11	1.595 (3)	C36—C53	1.510 (6)
P4—F13	1.600 (4)	C37—H37	0.95
P4—F8	1.598 (4)	C38—C39	1.522 (6)
P4—F10	1.604 (3)	C38—H38A	0.99
N12—C37	1.337 (5)	C38—H38B	0.99
N12—C17	1.368 (5)	C39—C16^i^	1.532 (6)
C14—C33	1.383 (6)	C39—H39A	0.99
C14—C34	1.386 (6)	C39—H39B	0.99
C15—C25	1.406 (6)	C40—H40A	0.98
C15—C35	1.422 (6)	C40—H40B	0.98
C15—C20	1.428 (6)	C40—H40C	0.98
C16—C18	1.528 (6)	C41—C45	1.394 (6)
C16—C39^i^	1.532 (6)	C42—C50	1.391 (7)
C16—H16A	0.99	C43—C54	1.498 (6)
C16—H16B	0.99	C43—H43A	0.98
C17—C30	1.406 (6)	C43—H43B	0.98
C17—C25	1.446 (5)	C43—H43C	0.98
C18—H18A	0.99	C44—C51	1.372 (7)
C18—H18B	0.99	C44—C52	1.374 (7)
N19—C49	1.330 (5)	C44—H44	0.95
N19—C25	1.357 (5)	C45—C55	1.396 (6)
C20—C22	1.383 (6)	C45—H45	0.95
C20—C27	1.507 (6)	C46—C47	1.376 (9)
C21—C42	1.387 (6)	C46—H46	0.95
C21—C24	1.398 (7)	C47—C57	1.350 (9)
C21—H21	0.95	C47—H47	0.95
C22—C49	1.399 (6)	C48—H48	0.95
C22—C40	1.505 (6)	C49—H49	0.95
C23—C48	1.376 (7)	C50—C59	1.391 (7)
C23—C41	1.406 (6)	C50—H50	0.95
C23—H23	0.95	C51—H51	0.95
C24—C56	1.379 (9)	C52—H52	0.95
C24—H24	0.95	C53—H53A	0.98
C26—C46	1.392 (8)	C53—H53B	0.98
C26—C29	1.395 (6)	C53—H53C	0.98
C26—H26	0.95	C55—H55	0.95
C27—H27A	0.98	C56—C59	1.360 (9)
C27—H27B	0.98	C56—H56	0.95
C27—H27C	0.98	C57—H57	0.95
C28—C35	1.356 (6)	C59—H59	0.95
C28—C30	1.436 (6)	Cl1—C100	1.751 (6)
C28—H28	0.95	Cl2—C100	1.764 (6)
C29—C32	1.380 (6)	C100—H10A	0.99
C30—C54	1.423 (6)	C100—H10B	0.99
			
N12—Cu1—N19	80.10 (13)	C57—C32—H32	119.3
N12—Cu1—P2	127.97 (10)	C14—C33—C51	120.2 (4)
N19—Cu1—P2	111.67 (13)	C14—C33—H33	119.9
N12—Cu1—P3	100.49 (11)	C51—C33—H33	119.9
N19—Cu1—P3	104.06 (12)	C14—C34—C52	120.1 (4)
P2—Cu1—P3	122.83 (8)	C14—C34—H34	119.9
C41—P2—C38	105.3 (2)	C52—C34—H34	119.9
C41—P2—C14	102.2 (2)	C28—C35—C15	121.6 (4)
C38—P2—C14	102.7 (2)	C28—C35—H35	119.2
C41—P2—Cu1	106.99 (16)	C15—C35—H35	119.2
C38—P2—Cu1	118.10 (16)	C54—C36—C37	119.1 (4)
C14—P2—Cu1	119.70 (16)	C54—C36—C53	122.3 (4)
C29—P3—C18	103.9 (2)	C37—C36—C53	118.5 (4)
C29—P3—C42	105.8 (2)	N12—C37—C36	124.1 (4)
C18—P3—C42	103.2 (2)	N12—C37—H37	118
C29—P3—Cu1	109.29 (15)	C36—C37—H37	118
C18—P3—Cu1	119.27 (14)	C39—C38—P2	110.2 (3)
C42—P3—Cu1	114.08 (15)	C39—C38—H38A	109.6
F7—P4—F9	179.5 (2)	P2—C38—H38A	109.6
F7—P4—F11	89.82 (19)	C39—C38—H38B	109.6
F9—P4—F11	90.4 (2)	P2—C38—H38B	109.6
F7—P4—F13	89.8 (2)	H38A—C38—H38B	108.1
F9—P4—F13	89.8 (2)	C38—C39—C16^i^	112.9 (3)
F11—P4—F13	90.7 (2)	C38—C39—H39A	109
F7—P4—F8	91.1 (2)	C16^i^—C39—H39A	109
F9—P4—F8	89.4 (2)	C38—C39—H39B	109
F11—P4—F8	90.3 (2)	C16^i^—C39—H39B	109
F13—P4—F8	178.68 (19)	H39A—C39—H39B	107.8
F7—P4—F10	89.44 (18)	C22—C40—H40A	109.5
F9—P4—F10	90.3 (2)	C22—C40—H40B	109.5
F11—P4—F10	179.1 (2)	H40A—C40—H40B	109.5
F13—P4—F10	89.8 (2)	C22—C40—H40C	109.5
F8—P4—F10	89.21 (19)	H40A—C40—H40C	109.5
C37—N12—C17	117.3 (4)	H40B—C40—H40C	109.5
C37—N12—Cu1	128.3 (3)	C45—C41—C23	118.5 (4)
C17—N12—Cu1	112.2 (3)	C45—C41—P2	120.7 (3)
C33—C14—C34	119.2 (4)	C23—C41—P2	120.3 (3)
C33—C14—P2	119.1 (3)	C50—C42—C21	118.6 (4)
C34—C14—P2	121.7 (3)	C50—C42—P3	115.8 (3)
C25—C15—C35	118.2 (4)	C21—C42—P3	125.5 (4)
C25—C15—C20	117.6 (4)	C54—C43—H43A	109.5
C35—C15—C20	124.2 (4)	C54—C43—H43B	109.5
C18—C16—C39^i^	113.3 (3)	H43A—C43—H43B	109.5
C18—C16—H16A	108.9	C54—C43—H43C	109.5
C39^i^—C16—H16A	108.9	H43A—C43—H43C	109.5
C18—C16—H16B	108.9	H43B—C43—H43C	109.5
C39^i^—C16—H16B	108.9	C51—C44—C52	120.1 (4)
H16A—C16—H16B	107.7	C51—C44—H44	120
N12—C17—C30	122.7 (4)	C52—C44—H44	120
N12—C17—C25	116.8 (4)	C55—C45—C41	120.4 (4)
C30—C17—C25	120.5 (4)	C55—C45—H45	119.8
C16—C18—P3	115.4 (3)	C41—C45—H45	119.8
C16—C18—H18A	108.4	C47—C46—C26	121.3 (5)
P3—C18—H18A	108.4	C47—C46—H46	119.3
C16—C18—H18B	108.4	C26—C46—H46	119.3
P3—C18—H18B	108.4	C57—C47—C46	119.4 (5)
H18A—C18—H18B	107.5	C57—C47—H47	120.3
C49—N19—C25	117.4 (4)	C46—C47—H47	120.3
C49—N19—Cu1	129.5 (3)	C23—C48—C31	120.7 (5)
C25—N19—Cu1	111.7 (2)	C23—C48—H48	119.7
C22—C20—C15	119.2 (4)	C31—C48—H48	119.7
C22—C20—C27	120.1 (4)	N19—C49—C22	124.8 (4)
C15—C20—C27	120.7 (4)	N19—C49—H49	117.6
C42—C21—C24	120.7 (5)	C22—C49—H49	117.6
C42—C21—H21	119.7	C42—C50—C59	120.4 (5)
C24—C21—H21	119.7	C42—C50—H50	119.8
C20—C22—C49	117.9 (4)	C59—C50—H50	119.8
C20—C22—C40	124.0 (4)	C44—C51—C33	120.2 (4)
C49—C22—C40	118.1 (4)	C44—C51—H51	119.9
C48—C23—C41	120.5 (4)	C33—C51—H51	119.9
C48—C23—H23	119.8	C44—C52—C34	120.0 (4)
C41—C23—H23	119.8	C44—C52—H52	120
C56—C24—C21	119.4 (5)	C34—C52—H52	120
C56—C24—H24	120.3	C36—C53—H53A	109.5
C21—C24—H24	120.3	C36—C53—H53B	109.5
N19—C25—C15	123.0 (4)	H53A—C53—H53B	109.5
N19—C25—C17	116.8 (3)	C36—C53—H53C	109.5
C15—C25—C17	120.2 (4)	H53A—C53—H53C	109.5
C46—C26—C29	119.2 (5)	H53B—C53—H53C	109.5
C46—C26—H26	120.4	C36—C54—C30	118.1 (4)
C29—C26—H26	120.4	C36—C54—C43	119.8 (4)
C20—C27—H27A	109.5	C30—C54—C43	122.1 (4)
C20—C27—H27B	109.5	C31—C55—C45	120.2 (5)
H27A—C27—H27B	109.5	C31—C55—H55	119.9
C20—C27—H27C	109.5	C45—C55—H55	119.9
H27A—C27—H27C	109.5	C59—C56—C24	120.5 (5)
H27B—C27—H27C	109.5	C59—C56—H56	119.7
C35—C28—C30	122.3 (4)	C24—C56—H56	119.7
C35—C28—H28	118.8	C47—C57—C32	120.4 (6)
C30—C28—H28	118.8	C47—C57—H57	119.8
C32—C29—C26	118.2 (4)	C32—C57—H57	119.8
C32—C29—P3	120.9 (3)	C56—C59—C50	120.4 (6)
C26—C29—P3	120.3 (4)	C56—C59—H59	119.8
C17—C30—C54	118.6 (4)	C50—C59—H59	119.8
C17—C30—C28	117.2 (4)	Cl1—C100—Cl2	110.9 (3)
C54—C30—C28	124.2 (4)	Cl1—C100—H10A	109.5
C55—C31—C48	119.7 (5)	Cl2—C100—H10A	109.5
C55—C31—H31	120.1	Cl1—C100—H10B	109.5
C48—C31—H31	120.1	Cl2—C100—H10B	109.5
C29—C32—C57	121.4 (5)	H10A—C100—H10B	108.1
C29—C32—H32	119.3		
			
N12—Cu1—P2—C41	−124.73 (19)	Cu1—P3—C29—C26	80.0 (4)
N19—Cu1—P2—C41	−30.41 (18)	N12—C17—C30—C54	−1.3 (6)
P3—Cu1—P2—C41	94.16 (16)	C25—C17—C30—C54	176.8 (4)
N12—Cu1—P2—C38	116.9 (2)	N12—C17—C30—C28	−179.9 (4)
N19—Cu1—P2—C38	−148.81 (18)	C25—C17—C30—C28	−1.8 (6)
P3—Cu1—P2—C38	−24.24 (16)	C35—C28—C30—C17	2.7 (6)
N12—Cu1—P2—C14	−9.4 (2)	C35—C28—C30—C54	−175.8 (4)
N19—Cu1—P2—C14	84.91 (19)	C26—C29—C32—C57	0.4 (7)
P3—Cu1—P2—C14	−150.52 (16)	P3—C29—C32—C57	171.5 (4)
N12—Cu1—P3—C29	−6.90 (17)	C34—C14—C33—C51	2.6 (7)
N19—Cu1—P3—C29	−89.21 (18)	P2—C14—C33—C51	−177.2 (4)
P2—Cu1—P3—C29	142.87 (16)	C33—C14—C34—C52	−1.3 (7)
N12—Cu1—P3—C18	−126.11 (19)	P2—C14—C34—C52	178.5 (4)
N19—Cu1—P3—C18	151.59 (18)	C30—C28—C35—C15	−1.0 (6)
P2—Cu1—P3—C18	23.67 (17)	C25—C15—C35—C28	−1.6 (6)
N12—Cu1—P3—C42	111.35 (18)	C20—C15—C35—C28	178.6 (4)
N19—Cu1—P3—C42	29.04 (19)	C17—N12—C37—C36	−2.1 (6)
P2—Cu1—P3—C42	−98.87 (18)	Cu1—N12—C37—C36	−164.0 (3)
N19—Cu1—N12—C37	175.8 (4)	C54—C36—C37—N12	0.7 (6)
P2—Cu1—N12—C37	−74.4 (4)	C53—C36—C37—N12	178.9 (4)
P3—Cu1—N12—C37	73.2 (4)	C41—P2—C38—C39	166.2 (3)
N19—Cu1—N12—C17	13.1 (3)	C14—P2—C38—C39	59.6 (3)
P2—Cu1—N12—C17	122.9 (3)	Cu1—P2—C38—C39	−74.5 (3)
P3—Cu1—N12—C17	−89.5 (3)	P2—C38—C39—C16^i^	176.9 (3)
C41—P2—C14—C33	118.2 (4)	C48—C23—C41—C45	1.3 (6)
C38—P2—C14—C33	−132.8 (4)	C48—C23—C41—P2	−170.4 (4)
Cu1—P2—C14—C33	0.4 (4)	C38—P2—C41—C45	47.9 (4)
C41—P2—C14—C34	−61.6 (4)	C14—P2—C41—C45	154.9 (4)
C38—P2—C14—C34	47.4 (4)	Cu1—P2—C41—C45	−78.5 (4)
Cu1—P2—C14—C34	−179.4 (3)	C38—P2—C41—C23	−140.5 (3)
C37—N12—C17—C30	2.3 (6)	C14—P2—C41—C23	−33.6 (4)
Cu1—N12—C17—C30	167.1 (3)	Cu1—P2—C41—C23	93.0 (3)
C37—N12—C17—C25	−175.8 (3)	C24—C21—C42—C50	−1.6 (7)
Cu1—N12—C17—C25	−11.0 (4)	C24—C21—C42—P3	−179.9 (3)
C39^i^—C16—C18—P3	−176.9 (3)	C29—P3—C42—C50	157.2 (4)
C29—P3—C18—C16	61.4 (3)	C18—P3—C42—C50	−93.9 (4)
C42—P3—C18—C16	−48.9 (3)	Cu1—P3—C42—C50	37.0 (4)
Cu1—P3—C18—C16	−176.6 (2)	C29—P3—C42—C21	−24.4 (4)
N12—Cu1—N19—C49	−179.5 (4)	C18—P3—C42—C21	84.5 (4)
P2—Cu1—N19—C49	53.4 (4)	Cu1—P3—C42—C21	−144.6 (3)
P3—Cu1—N19—C49	−81.1 (4)	C23—C41—C45—C55	−0.4 (7)
N12—Cu1—N19—C25	−13.4 (3)	P2—C41—C45—C55	171.2 (4)
P2—Cu1—N19—C25	−140.4 (2)	C29—C26—C46—C47	0.4 (7)
P3—Cu1—N19—C25	85.1 (3)	C26—C46—C47—C57	−0.2 (8)
C25—C15—C20—C22	−0.7 (6)	C41—C23—C48—C31	−0.6 (7)
C35—C15—C20—C22	179.1 (4)	C55—C31—C48—C23	−0.9 (8)
C25—C15—C20—C27	178.0 (4)	C25—N19—C49—C22	−2.8 (6)
C35—C15—C20—C27	−2.3 (6)	Cu1—N19—C49—C22	162.7 (3)
C15—C20—C22—C49	−0.3 (6)	C20—C22—C49—N19	2.1 (6)
C27—C20—C22—C49	−179.0 (4)	C40—C22—C49—N19	−176.4 (4)
C15—C20—C22—C40	178.1 (4)	C21—C42—C50—C59	1.6 (7)
C27—C20—C22—C40	−0.6 (7)	P3—C42—C50—C59	−179.9 (4)
C42—C21—C24—C56	0.3 (7)	C52—C44—C51—C33	−1.8 (8)
C49—N19—C25—C15	1.6 (6)	C14—C33—C51—C44	−1.1 (7)
Cu1—N19—C25—C15	−166.3 (3)	C51—C44—C52—C34	3.1 (8)
C49—N19—C25—C17	179.6 (3)	C14—C34—C52—C44	−1.5 (7)
Cu1—N19—C25—C17	11.6 (4)	C37—C36—C54—C30	0.4 (6)
C35—C15—C25—N19	−179.7 (3)	C53—C36—C54—C30	−177.7 (4)
C20—C15—C25—N19	0.0 (6)	C37—C36—C54—C43	179.9 (4)
C35—C15—C25—C17	2.4 (6)	C53—C36—C54—C43	1.7 (7)
C20—C15—C25—C17	−177.8 (3)	C17—C30—C54—C36	−0.1 (6)
N12—C17—C25—N19	−0.5 (5)	C28—C30—C54—C36	178.3 (4)
C30—C17—C25—N19	−178.7 (3)	C17—C30—C54—C43	−179.6 (4)
N12—C17—C25—C15	177.5 (3)	C28—C30—C54—C43	−1.1 (7)
C30—C17—C25—C15	−0.7 (6)	C48—C31—C55—C45	1.8 (8)
C46—C26—C29—C32	−0.5 (6)	C41—C45—C55—C31	−1.1 (8)
C46—C26—C29—P3	−171.7 (4)	C21—C24—C56—C59	1.1 (8)
C18—P3—C29—C32	37.4 (4)	C46—C47—C57—C32	0.0 (8)
C42—P3—C29—C32	145.8 (3)	C29—C32—C57—C47	−0.1 (8)
Cu1—P3—C29—C32	−91.0 (3)	C24—C56—C59—C50	−1.1 (9)
C18—P3—C29—C26	−151.7 (3)	C42—C50—C59—C56	−0.2 (9)
C42—P3—C29—C26	−43.3 (4)		

Symmetry code: (i) −*x*, −*y*, −*z*.

### Hydrogen-bond geometry (Å, º)

**Table:**

*D*—H···*A*	*D*—H	H···*A*	*D*···*A*	*D*—H···*A*
C32—H32···F10^ii^	0.95	2.51	3.382 (6)	152
C100—H10*A*···F11^iii^	0.99	2.39	3.360 (8)	165
C100—H10*A*···F13^iii^	0.99	2.55	3.373 (9)	141

Symmetry codes: (ii) −*x*, −*y*, −*z*+1; (iii) *x*+1, *y*, *z*.
